# Towards a bioengineered uterus: bioactive sheep uterus scaffolds are effectively recellularized by enzymatic preconditioning

**DOI:** 10.1038/s41536-021-00136-0

**Published:** 2021-05-21

**Authors:** Arvind Manikantan Padma, Laura Carrière, Frida Krokström Karlsson, Edina Sehic, Sara Bandstein, Tom Tristan Tiemann, Mihai Oltean, Min Jong Song, Mats Brännström, Mats Hellström

**Affiliations:** 1 Laboratory for Transplantation and Regenerative Medicine, Sahlgrenska Academy, University of Gothenburg, Gothenburg, Sweden; 2 Department of Obstetrics and Gynecology, Clinical Sciences, Sahlgrenska Academy, University of Gothenburg, Gothenburg, Sweden; 3 Department of Gynecology and Obstetrics, University Hospital of Heidelberg, Heidelberg, Germany; 4 Department of Surgery, Clinical Sciences, Sahlgrenska Academy, University of Gothenburg, Gothenburg, Sweden; 5 Department of Obstetrics and Gynecology, Yeouido St. Mary’s Hospital, The Catholic University of Korea, Seoul, Republic of Korea; 6 Stockholm IVF-EUGIN, Hammarby allé 93, Stockholm, Sweden

**Keywords:** Preclinical research, Regeneration, Tissue engineering, Regenerative medicine

## Abstract

Uterine factor infertility was considered incurable until recently when we reported the first successful live birth after uterus transplantation. However, risky donor surgery and immunosuppressive therapy are factors that may be avoided with bioengineering. For example, transplanted recellularized constructs derived from decellularized tissue restored fertility in rodent models and mandate translational studies. In this study, we decellularized whole sheep uterus with three different protocols using 0.5% sodium dodecyl sulfate, 2% sodium deoxycholate (SDC) or 2% SDC, and 1% Triton X-100. Scaffolds were then assessed for bioactivity using the dorsal root ganglion and chorioallantoic membrane assays, and we found that all the uterus scaffolds exhibited growth factor activity that promoted neurogenesis and angiogenesis. Extensive recellularization optimization was conducted using multipotent sheep fetal stem cells and we report results from the following three in vitro conditions; (a) standard cell culturing conditions, (b) constructs cultured in transwells, and (c) scaffolds preconditioned with matrix metalloproteinase 2 and 9. The recellularization efficiency was improved short-term when transwells were used compared with standard culturing conditions. However, the recellularization efficiency in scaffolds preconditioned with matrix metalloproteinases was 200–300% better than the other strategies evaluated herein, independent of decellularization protocol. Hence, a major recellularization hurdle has been overcome with the improved recellularization strategies and in vitro platforms described herein. These results are an important milestone and should facilitate the production of large bioengineered grafts suitable for future in vivo applications in the sheep, which is an essential step before considering these principles in a clinical setting.

## Introduction

Infertility, caused by an absent or dysfunctional uterus was considered incurable until our group succeeded with the world’s first uterus transplantation that resulted in live birth^1^. This procedure has now been repeated at multiple centers resulting in more reported births^2–9^. Uterus transplantation is still considered an experimental treatment procedure with protocols continuously being evaluated, i.e., organ preservation protocols^10^, positioning of the vascular anastomoses^11,12^, and the use of robotic-assisted surgery to reduce recovery time and blood loss^13–15^. So far, almost all successful uterus transplantation cases have used live donors. However, the risks entailed by live donor surgery and the negative side-effects caused by the required immunosuppressive treatment in the allograft recipient opens up discussions for an alternative donor source.

An attractive organ option which would overcome some of these limitations is a developed bioengineered uterus from an appropriate scaffold together with the patient’s own cells. Extracellular matrix (ECM) derived scaffolds proved successful in several bioengineering studies on female reproductive tissues^16–20^. Recently, ECM-derived uterus scaffolds were created by decellularization for the mouse^21^, the rat^22–26^, the rabbit^27^, the pig^28^, and more recently, the sheep^29,30^. Furthermore, a series of successful studies on rodents from multiple independent groups suggest that decellularized uterine tissue patches can be grafted to repair a full-thickness uterine wall injury and restore fertility^21,24,26,31^. These successful results mandate a translational evaluation using larger animal models. We therefore developed decellularization protocols for the generation of sheep uterus scaffolds as the first step in this process^30^. The sheep is by many considered the most suitable preclinical large animal model for uterine studies because of its physiological and anatomical resemblance to the human uterus, and the sheep model played a significant part in the lead up to the successful human uterus transplantation cases^32,33^.

However, a major obstacle using decellularized tissue for bioengineering applications is the limited ability to recellularize the constructs and achieve an appropriate cell density which seems to play an important role in the treatment outcome. For example, recellularized constructs seem to better stimulate tissue regeneration, recruit host cells and prevent rapid scaffold degeneration compared to scaffolds without cells, in particular for larger grafts^34–37^.

We herein studied three different types of sheep uterus scaffolds created by decellularization, and assessed their ability to stimulate growth and angiogenesis. We further describe novel strategies how to significantly increase the recellularization efficiency. This study is therefore an important in vitro milestone toward sheep uterus bioengineering in vivo.

## Results

### Sheep uterus scaffold generation

**Fig. 1:**
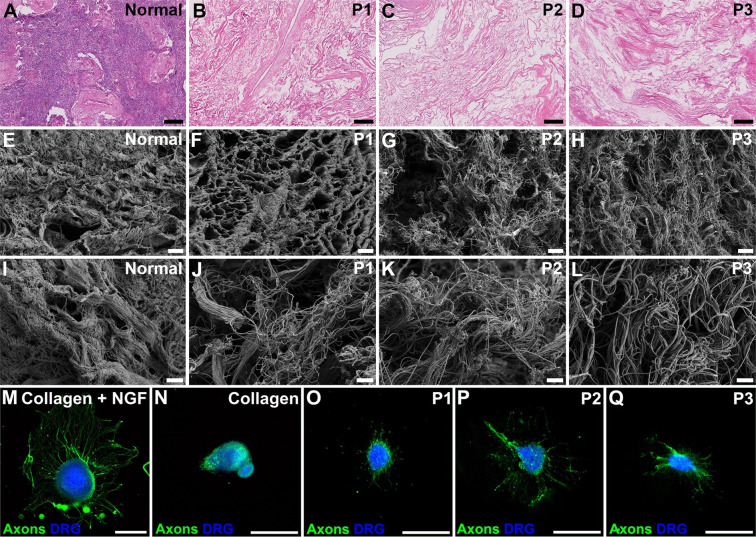
Morphological and bioactivity assessment of developed sheep uterus scaffolds. Hematoxylin and eosin-stained sections from normal and decellularized uterus showed that the extracellular matrix was well-preserved and that all hematoxylin positive nuclei had been removed by the different decellularization protocols (**A**–**D**; scale bar = 100 μm). Scanning electron microscopy pictures obtained at ×3000 magnification (**E**–**H**; scale bar = 8 μm) showed that protocol (P) 1 generated scaffolds resulted in an organized porous structure (**F**) while P2 and P3 derived scaffolds resulted in a more compact arrangement with less organized fiber structure. Higher magnification (×12,000; **I**–**L**; scale bar = 2 μm) showed that collagen bundles remained intact in all three scaffold types. The dorsal root ganglion (DRG) assay and the chorioallantioc membrane (CAM) assay showed that all scaffolds were functionally bioactive (**M**–**V**). The DRGs regenerated axons in wells coated with collagen that was given nerve growth factor supplement in the culture medium (positive control; **M**), but not when this growth factor was omitted (negative control, **N**). Scaffolds derived from the three different decellularization protocols all stimulated axonal regeneration (**O**–**Q**; scale bar = 500 µm).

Whole sheep uterus organs were decellularized using three earlier established protocols^30^. Based on microscopic analyses, we were able to reproduce the successful protocols as no donor cells were observed. Protocol 1 (P1) derived scaffolds based on sodium dodecyl sulfate (SDS) appeared denser and demonstrated a superior morphological ECM fiber structure compared with protocol 2 (P2) generated scaffolds that was based on sodium deoxycholeate (SDC) or to scaffolds produced by protocol 3 (P3) that was based on a combination of SDC and Triton X-100. Higher magnification in the deeper layer of the myometrium compartment in scanning electron microscopy on cross sectioned dUTDs revealed more abundant collagen bundles in P1 and P2 derived scaffolds, while scaffolds generated with P3 seemed more porous (Fig. 1A–L).

### Scaffold bioactivity induced axon regeneration

The bioactivity of the decellularized sheep uteri was assessed using the fetal dorsal root ganglion (DRG) assay that detects neurogenesis. It was evident that the decellularized sheep uterus tissue discs (dUTDs) displayed functional growth-promoting qualities that stimulated more axon regeneration to longer distances compared to collagen alone (Fig. 1M–Q).

### Scaffold preconditioning with MMPs

**Fig. 2:**
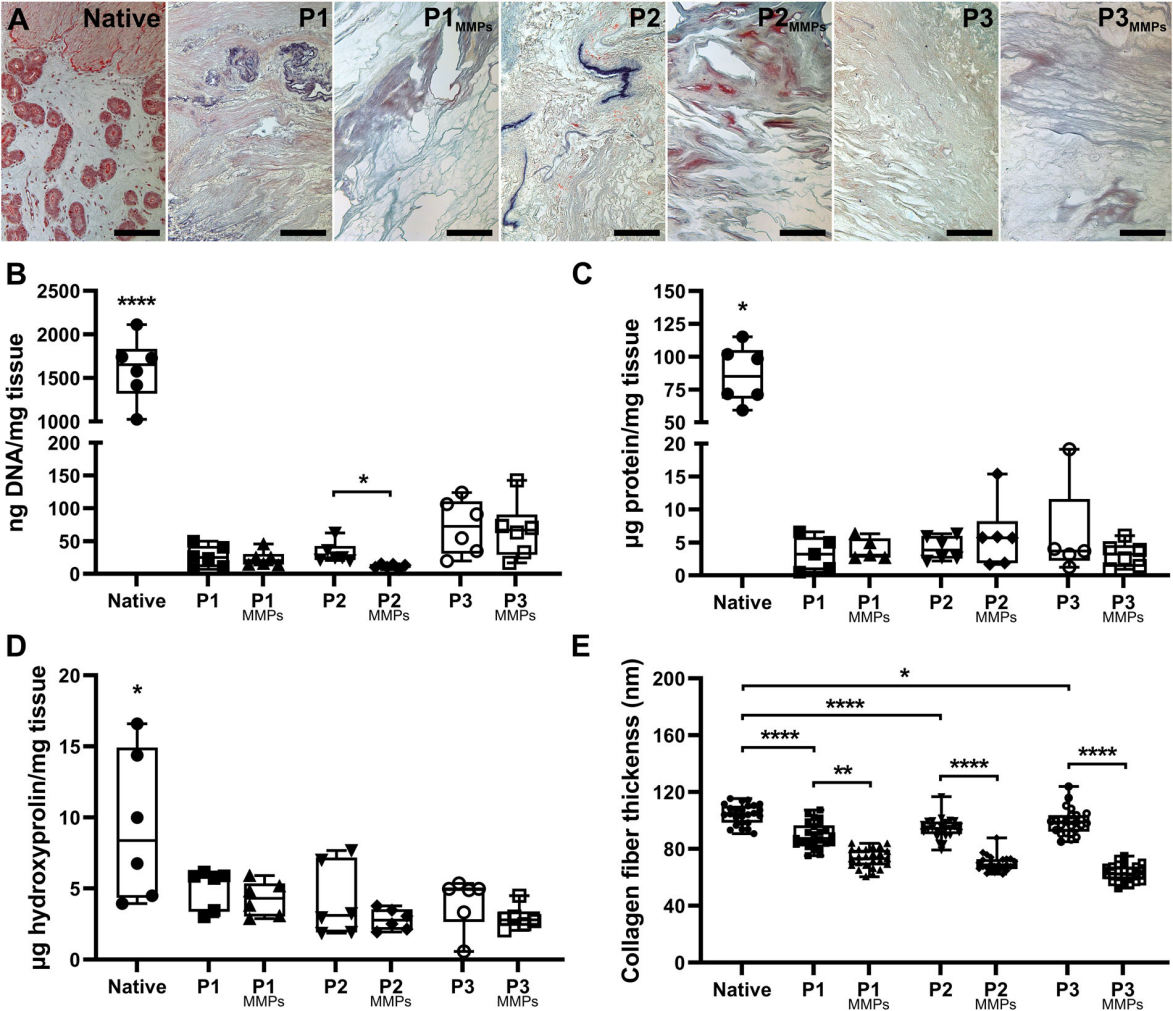
The extracellular matrix composition of developed scaffolds was assessed. **A** Russell-Movat pentachrome stains collagen and reticular fibers (yellow), nuclei and elastic fibers (black), sulfated glycosaminoglycans (blue), muscle tissue (red), and fibrin intense red. Stained from scaffolds revealed that the matrix metalloproteinase 2 and 9 (MMPs) treatment resulted in a more porous scaffold and that elastic fibers became more distinct. Scale bars = 100 µm. **B** Each decellularization method resulted in scaffolds with very small amounts of remaining donor DNA, and that the MMPs were able to reduce this even more in protocol (P) 2 derived scaffolds. **C** The total protein content and **D** the hydroxyproline content were unaffected by the MMPs treatment while **E** the collagen fiber thickness was significantly reduced. Graphs show box plot with each sample value, median ± IQR, and range.

**Fig. 3:**
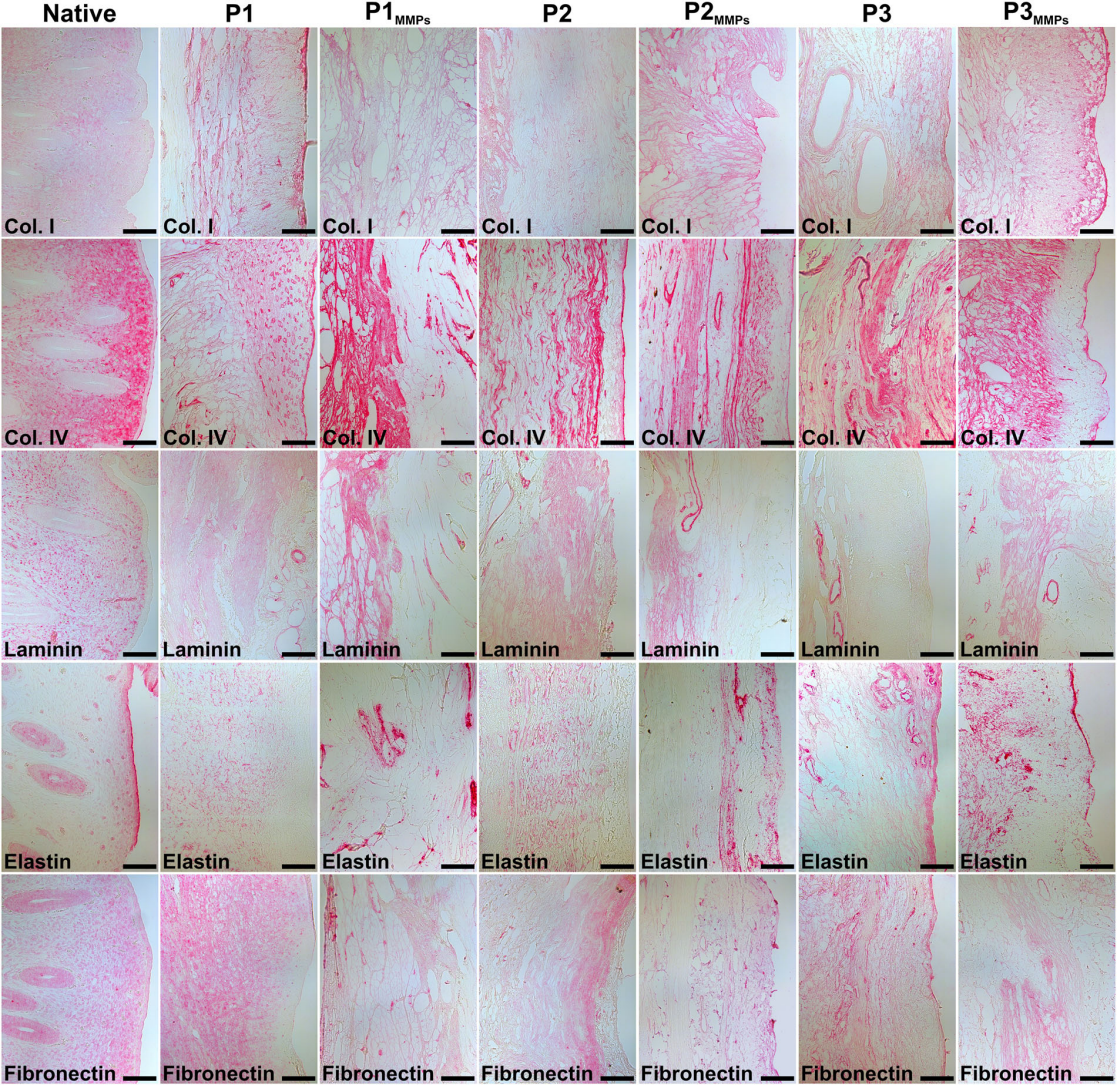
Distribution of importantant structural components after scaffold production. Immunohistochemistry stained sections from decellularized sheep uterus tissue before and after matrix metalloproteinase 2 and 9 (MMPs) preconditioning, including native uterus tissue for comparison. P protocol, Col. collagen. Scale bars = 100 µm.

To evaluate if improved cell attachment and scaffold porosity could be established to increase future scaffold recellularization efficiencies, we preconditioned dUTDs with matrix metalloproteinases (MMPs) 2 and 9. Morphologically, the tissue became more porous after the MMPs treatment and there were more distinctly stained elastic fibers after the preconditioning. Few other differences were observed in Russel-Movat pentachrome stained sections before and after MMPs treatment (Fig. 2A). Importantly, the MMPs treatment did not affect the vascular patency in large uterus scaffolds, as confirmed by vascular perfusion (Supplementary videos 1–3). Scaffold content of donor DNA, total protein, and hydroxyproline were also unaffected from the preconditioning procedure (Fig. 2B–D). The actual decellularization process did not affect the collagen fiber thickness. However, the MMPs treatment reduced it by 30–40% in all three scaffold types (Fig. 2E). Immunohistochemistry for collagen I, collagen IV, laminin, elastin, and fibronectin showed that these molecules were well retained in the scaffolds, but elastin had aggregated to specific areas within the scaffolds after the enzymatic treatment (Fig. 3).

### Confirmed angiogenic bioactivity in all scaffold types

**Fig. 4:**
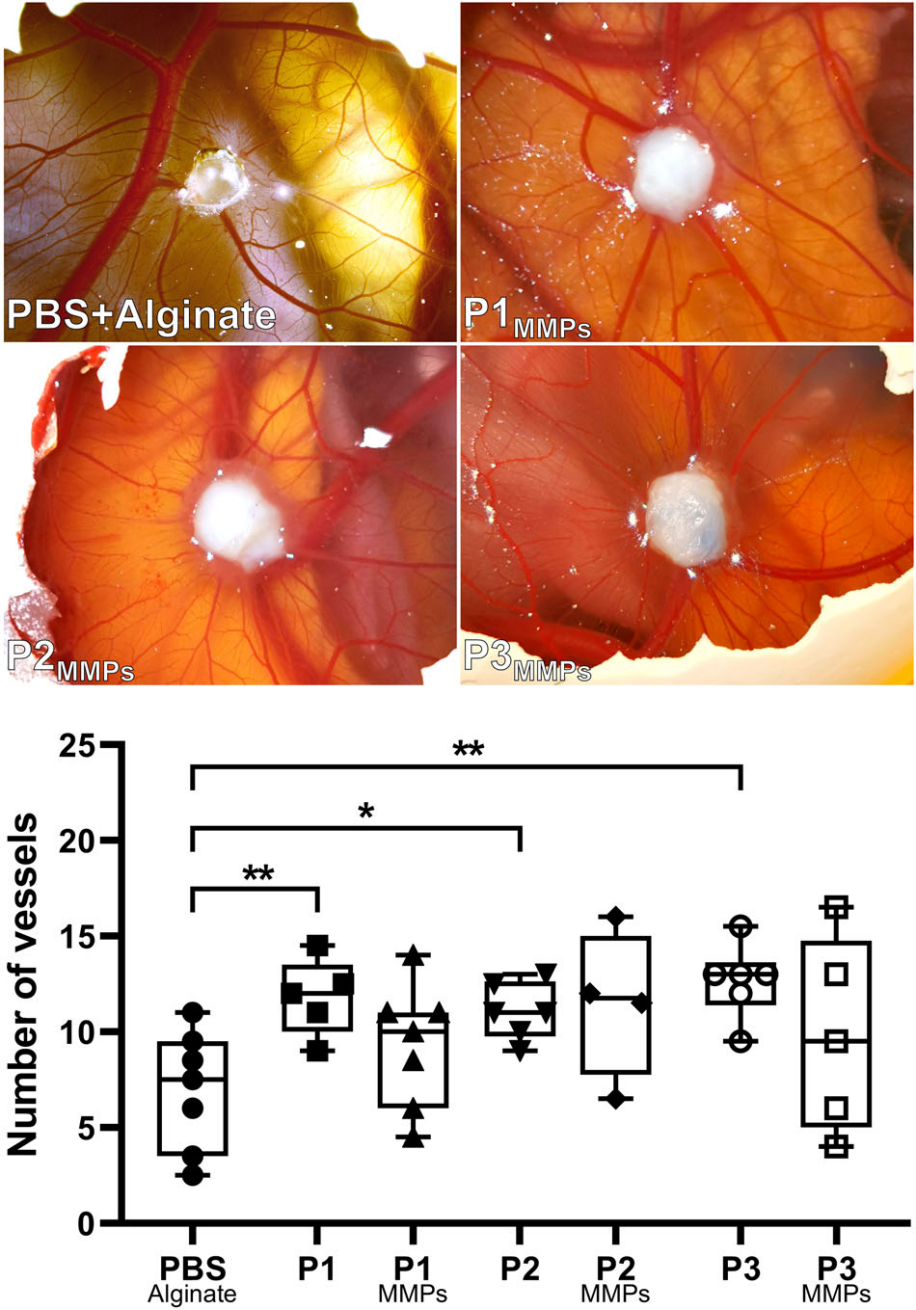
The chorioallantioc membrane (CAM) bioactivity assay confirmed a significant increase in angiogenesis after the inoculation of the three scaffold types before the matrix metalloproteinase 2 and 9 (MMPs) preconditioning compared with the inert alginate reference point that indicate normal chick fetal angiogenesis. The bioactivity from the scaffolds stimulated a doubling of blood vessel formation, while there was a modest (non-significant) reduction in angiogenic effects after the MMPs treatment. Graphs show box plot with each sample value, median ± IQR, and range. P protocol. Significant levels; *p < 0.05; **p < 0.01.

The scaffolds were able to actively stimulate angiogenesis, evident by the CAM assay. Each scaffold type resulted in a near doubling of blood vessels in the vicinity of the inoculated decellularized uterus tissue when compared with the inert control reference point that does not stimulate any extra angiogenesis (Fig. 4). There was no difference in angiogenic effects between any of the uterus scaffold types before and after MMPs treatment.

### Cell characterization of SF-SCs

**Fig. 5:**
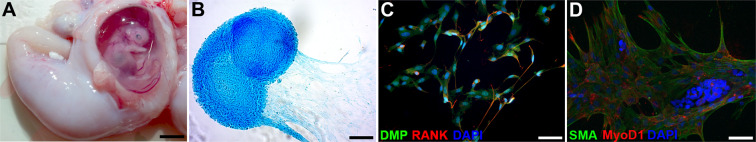
Pluripotent cells used for the recellularization. Heterogeneous sheep fetal stem cells (SF-SCs) were isolated from a 6–8-week-old sheep fetus (**A**). These cells were expanded in vitro and their pluripotency was demonstrated after their differentiation into a chondrogenic lineage (alcian blue positive clusters; **B**), an osteogenic lineage positive for dentin matrix acidic phosphoprotein 1 (DMP1) and receptor activator of nuclear factor κ B (RANK; **C**), and a myogenic lineage positive for smooth muscle cell actin (SMA) and myoblast determination protein 1 (MyoD1; **D**). Scale bars; 5 cm (**A**), and 100 μm (**B**–**D**).

Heterogeneous female sheep fetal bone marrow stem cells (SF-SC) were isolated from sheep fetus (Fig. 5A) and characterized for multipotency. The isolated SF-SCs were cuboidal and elongated under phase contrast microscope and were positive for the mesenchymal and stromal markers CD166 and vimentin (VIM), α-smooth muscle cell actin (SMA), and for estrogen receptor-β1 (ER-β; not shown). The multipotency was demonstrated by the differentiation into chondrogenic lineage with alcian blue positive cell clusters, osteogenic lineage (positive for dentin matrix acidic phosphoprotein 1, DMP and receptor activator of nuclear factor κ B, RANK), and myogenic lineage (positive for SMA and myoblast determination protein 1, MyoD1; Fig. 5).

### Recellularization efficiency using standard conditions

**Fig. 6:**
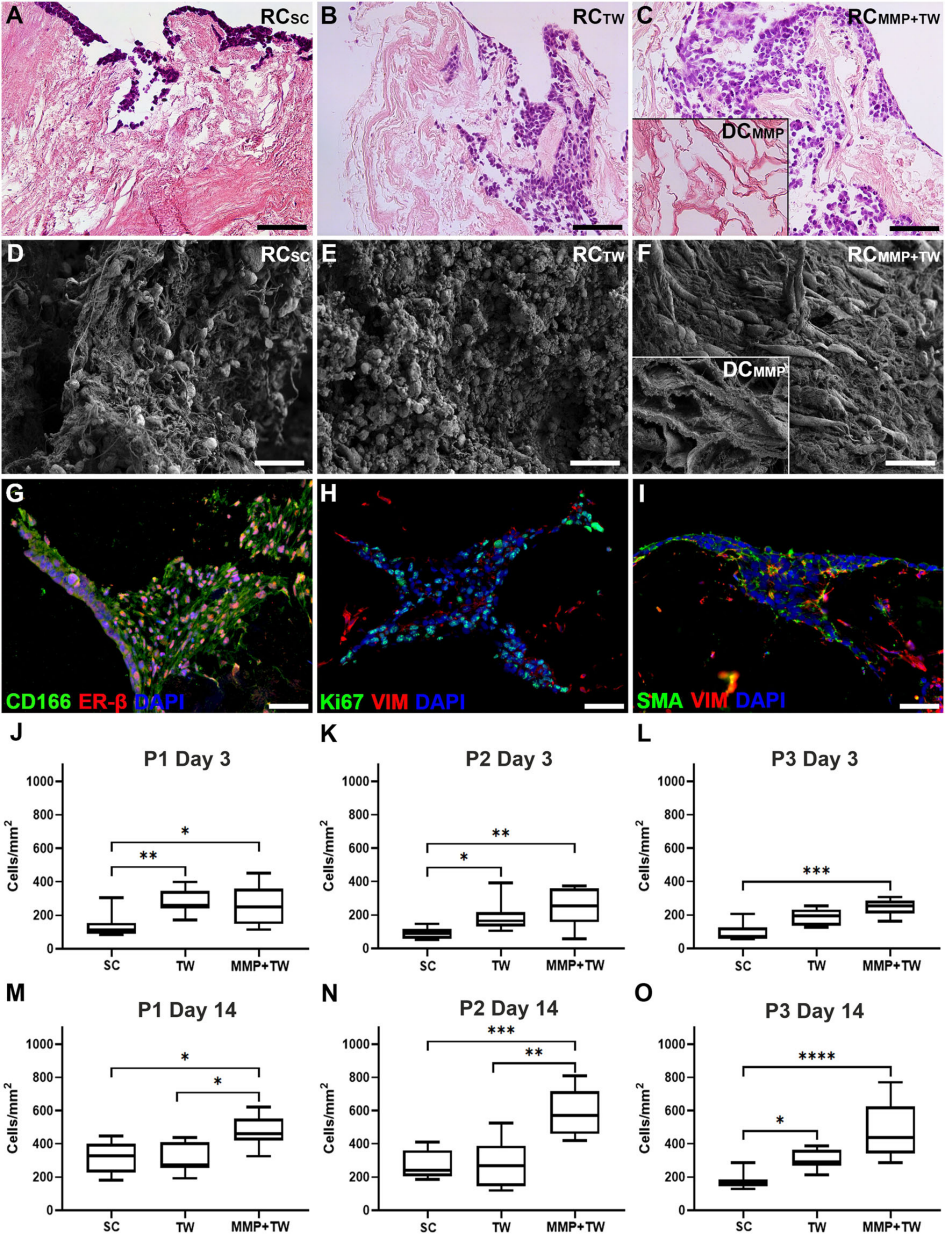
Scaffold preconditioning and transwells significantly improved the recellularization effiecinecy. Representative hematoxylin and eosin-stained sections of recellularized constructs from each culturing condition after 14 days in vitro (**A**–**C**; scale bar = 100 μm). Cells were mainly located around the injection site when standard culturing conditions (RC_SC_) were used and there was limited migration (**A**). However, the different recellularization strategies (transwells, RC_TW_) and matrix metalloproteinase enzyme preconditioning in combination with TW (RC_MMP+TW_) clearly improved the cell density and the cellular distribution throughout the scaffolding structure (**B**, **C**). Surface cell density was visualized by scanning electron microscope and showed a dense coverage, independent of strategy used (**D**–**F**; inserts in **C** and **F** show the scaffold preconditioned with MMPs without cells; DC_MMP_; scale bar = 20 μm). Sheep fetal stem cells kept their pluripotency during recellularization independent of scaffold type, evident by the continuation of CD166, estrogen receptor-β (ER-β), vimentin (VIM), α-smooth muscle actin (SMA), and Ki67 (proliferation) expression (**G**–**I**; scale bars=100μm). When the recellularization efficiency was quantified and compared between groups and recellularization strategy, it became obvious that preconditioning with MMPs and using transwells were advantageous already after 3 days in vitro (**J**–**L**), but a substantial improvement was seen after 14 days (**M**–**O**) in protocol 1 (P1), protocol 2 (P2), and protocols 3 (P3), respectively. Graphs show box plot with each sample value, median ± IQR, and range. Significant levels; *p < 0.05; **p < 0.01; ***p < 0.001, and ****p < 0.0001.

Cell counts from constructs recellularized with SF-SCs using standard conditions (RC_SC_) showed that the cell densities had significantly increased between day 3 and day 14 in all scaffold types (Table 1; Fig. 6). Sheep uterus scaffolds produced by P1 and P2 stimulated a greater cell proliferation during the extended cell culture period compared with scaffolds produced by P3 (Table 2). However, cells remained close to the injection site in all scaffold types and cell migration was only limited to include the superficial layers of the uterus scaffolds (Fig. 6A, D).

**Table 1:** Cell counts for each scaffold type and recellularization strategy used at respective time point after recellularization, including the calculated differences in *p*-value between relevant comparisons.

	Standard condition				Transwell				MMP + transwell		
	d3	d14	d3 vs. d14		d3	d14	d3 vs. d14		d3	d14	d3 vs. d14
	MED	MED	Difference		MED	MED	Difference		MED	MED	Difference
P1	112.8	328.7	*p* = 0.0003	P1	258.9	273.8	n.s.	P1	250.1	461.6	*p* = 0.0012
P2	88.7	240	*p* < 0.0001	P2	164.4	269.5	n.s.	P2	253.1	571.6	*p* < 0.0001
P3	69.8	165	*p* = 0.0076	P3	194.5	292.2	*p* = 0.0005	P3	253.1	437.9	*p* = 0.0002

**Table 2:** Statistical differences calculated based on the quantified recellularization efficiency (cell density) of different scaffold types (protocol 1, P1; protocol 2, P2; protocol 3, P3) compared with the same recellularization strategy and time point (d, days in vitro; MED, median; n.s., not significant).

Standard conditions			Transwell			MMP + transwell		
Difference	d3	d14	Difference	d3	d14	Difference	d3	d14
P1 vs. P2	n.s.	n.s.	P1 vs. P2	*p* = 0.0089	n.s.	P1 vs. P2	n.s.	n.s.
P1 vs. P3	n.s.	*p* = 0.0017	P1 vs. P3	*p* = 0.0411	n.s.	P1 vs. P3	n.s.	n.s.
P2 vs. P3	n.s.	*p* = 0.0156	P2 vs. P3	n.s.	n.s.	P2 vs. P3	n.s.	n.s.

### Improved short-term recellularization efficiency using transwells

To improve culture medium diffusion over the scaffold in vitro, recellularized constructs were cultured in transwell inserts (RC_TW_). Significantly more cells colonized P1 and P2 derived scaffolds compared with P3-derived scaffolds after 3 days in transwells (Table 2). This method was also better than RC_SC_ group after three days for P1 and P2 derived constructs (P1, *p* = 0.0074; P2, *p* = 0.0226) which represented a cell density increase of 229% and 185%, respectively. For P3-derived constructs, the efficiency increased by 278% using transwells (P3, *p* = 0.0504; Fig. 6J–L). P3, *p* = 0.050; Fig. 6J–L). Interestingly, there was no benefit using transwells when constructs were cultured for 14 days in vitro, except for P3-derived scaffolds (*p* = 0.0316; Fig. 6M–O). While we only detected a modest long-term cell density increase using transwells, we did observe improved cell migration using this method. Cells were found further away from the injection site compared with the RC_SC_ group (Fig. 6A, B).

### Enzymatic pre-treatment enhanced the recellularization efficiency

Indeed, preconditioned scaffolds with MMPs prior to the recellularization and subsequent transwell culture (RC_MMP+TW_) dramatically improved both cell density and cell distribution compared with the other two recellularization methods, which was further prominent after 14 days in vitro (Table 1; Fig. 6C, F). The significant increase in the recellularization efficiency after three days compared with RC_SC_ was 221%, 285%, and 363% for P1, P2, and P3, respectively (P1, *p* = 0.0293; P2, *p* = 0.0021; P3, *p* = 0.0002; Fig. 6J–L). The recellularization efficiency after 14 days in vitro and was significantly higher compared with 3e days after recellularization (Table 1). The improved cell density was also higher compared with RC_SC_ for all scaffold types (P1, *p* = 0.0108; P2, *p* = 0.0010; P3, *p* < 0.0001), and compared to methods using transwells without enzymatic pre-treatment for P1 (*p* = 0.0125) and P2 constructs (*p* = 0.0016), but not for the P3 constructs (Fig. 6M–O). Many cells were found in the peripheral parts of the constructs and it was evident that the enzymatic pre-treatment facilitated cell distribution across all scaffold compartments and generated better constructs than the other recellularization methods evaluated (Fig. 6A–C). The cells within the scaffolds remained positive for the stem cell phenotypic markers CD166, ER-β, α-SMA, and VIM during culture, and kept their proliferative ability, evident by Ki67 positive staining (Fig. 6G–I).

## Discussion

In initial studies on rat uterus bioengineering, we showed that scaffolds could be created by decellularization using SDC, DMSO, and Trirton X-100^22^. Simultaneously, two other groups reported that SDS or a hydrostatic pressure system could be used for the same purpose^24,26^. Together with transplantation studies, multiple reports clearly demonstrated successful bioengineering strategies using decellularized material to restore fertility in rodents with a uterine injury^21,23,31,38^. Similar whole uterus decellularization techniques were recently also developed and evaluated in vitro with tissues from larger animal models, e.g., the rabbit^27^, the pig^28^, and the sheep^29,30^.

However, it is evident that these scaffolds are difficult to recellularize efficiently. Thus, before conducting bioengineering studies in vivo on large animal models which will require significantly larger bioengineered constructs for engraftment, detailed assessments on scaffold quality and efficient recellularization methods need to be established since these factors play an essential role in graft functionality^34,39^. Effective recellularization of scaffolds depends on multiple factors, including the scaffold’s bioactivity and structural porosity that allow culture medium and cells to migrate across the construct during the reconstruction. We therefore investigated the functional bioactivity of three sheep uterine scaffolds and assessed their ability to stimulate growth and angiogenesis. We further assessed various methods to facilitate media access and construct porosity during recellularization with the hypothesis that they may improve cellular distribution across the construct and improve recellularization.

The first bioactivity test we conducted was the DRG assay. This method clearly demonstrated that all three decellularized uterus scaffolds stimulated axonal growth. This assay did not discriminate what specific growth factors were responsible for the beneficial neuronal sprouting. However, neurons are dependent on similar cell signaling growth pathways as other cell types^40^ and the positive effect seen on the DRGs therefore suggests the existence of beneficial cues for uterine tissue regeneration. For example, Hiraoka et al. showed that uterine tissue regeneration was interleukin-6 dependent after decellularized tissue was grafted in the mouse model^21^. This anti-inflammatory cytokine also acts as a powerful growth inducer in DRGs^41^. Nevertheless, additional studies will be required to determine what functional growth factors remained in the decellularized uterine tissue. However, this is complex since most straight-forward protein quantification methods are based on the detection of denatured protein fragments (i.e., non-functional proteins). In addition, our results also show that the scaffolds were free from detrimental remnants related to the decellularization procedures since it would be expected that neurons fail to initiate axonal sprouting if there were any remaining toxic decellularization chemicals^42^.

Similar to the DRG assay, the CAM assay confirmed functional bioactivity. This method determined scaffold-induced angiogenesis during the chicken embryo development and showed that our scaffolds stimulated a doubling of blood vessel formation on the CAM compared with normal angiogenesis during chicken fetus development, exemplified by the inert reference point used in the control group. Angiogenic activity will potentiate repair and reduce ischemic graft injury following engraftment. On the other hand, it still remains to be determined whether the effects seen are due to biologically active cues, or are due to physical/mechanical properties of the scaffolds^43^. The angiogenic effect was only mildly affected by the MMPs treatment. Histologically, we noticed that the MMPs treatment had affected the elastic fiber composition and the collagen fiber thickness while leaving the hydroxyproline content stable. Importantly, these structural changes did not affect the patency of the scaffolds’ vascular conduits. Hence, MMPs can be used as a scaffold preconditioning strategy in future experiments for developing larger constructs where vascular perfusion might be needed during whole organ recellularization and scaffold survival after engraftment (i.e., vascular anastomosis).

The physical properties of the scaffolds also affect cell attachment and recellularization. Recellularization is a challenging feat and few studies using decellularized tissue showed good cell densities. Furthermore, many bioengineering studies on assorted organs were transplanted after a relatively short time after recellularization^36,44–46^, including our own study in the rat^31^. In the latter study, we also reported that the cellular reconstruction is decellularization protocol-dependent and that it affected the outcomes in vivo. However, the choice of employed recellularization strategy also matters, concerning modes of cell delivery^47^, time cultured in vitro^48^ and type of in vitro platform used (e.g., various bioreactors or spinner flasks)^10,49–51^.

Hence, the experiments of the present study also aimed to evaluate novel in vitro strategies that may benefit recellularization. We first recellularized sheep uterus scaffolds using standard cell culturing conditions (the RC_SC_ group) which represents commonly employed recellularization strategies^28,30,31^. The results were comparable to earlier observations; cells were sparse and concentrated near the injection site and failed to migrate within the scaffolds.

We then evaluated an alternative in vitro setting, where recellularized scaffolds rested on a permeable membrane (a transwell), which would facilitate culture medium access (the RC_TW_ group). Surprisingly, this only provided a short-term benefit compared with the RC_SC_ group. Nevertheless, histological analysis indicated that cells had migrated better with this method, a result congruent with that of a study on kidney scaffold recellularization^52^.

We then assessed whether recellularization was improved further by the MMP2 and MMP9 preconditioning. We selected these enzymes because of their ability to specifically break down ECM components and because they are highly co-expressed by proliferative and migratory cells^53,54^ and can expedite angiogenesis^55^. The MMP pre-treatment was coupled with transwell cultures (RC_MMP+TW_ group), and the significant advantage was observed already after 3 days. Hence, this enzymatic preconditioning facilitated cell attachment. The effect was even more profound after 14 days when all constructs were colonized by several magnitudes higher cell numbers than after the other recellularization methods evaluated herein. Importantly, these results also showed that MMP treatment improved the least effective scaffold type (P3) to become as good as P1 and P2 derived scaffolds. Since no significant differences were seen between constructs after the enzymatic treatment, we suggest that a general reconditioning event took place that improved scaffold quality irrespective of decellularization protocol used. Furthermore, histology and scanning electron microscopy showed improved cell migration and distribution within all constructs after enzymatic preconditioning.

Naturally, the choice of cell type also plays a significant role in recellularization. It is likely necessary to use a combination of different cell types for bioengineering of large and more complex tissues than evaluated herein. However, we choose to use SF-SCs in this study that is a heterogeneous multipotent stroma cell population which could become valuable for future in vivo studies. A similar cell type was shown to produce important ECM components and growth factors that stimulate regeneration^53^. The SF-SCs continued to show markers for multipotentency and should thus still be able to activate valuable repair mechanisms after engraftment, e.g., as documented previously with multipotent stroma cells^56^ and in endometrial repair studies^57^. Future recellularization strategies may also include the use of hydrogels to prevent cell leakage after injection^47^, the use hypoxic in vitro conditions^28^, alternative preconditioning protocols, and more advanced cell culturing platforms.

In summary, this study demonstrated that our decellularized tissues were functionally bioactive and stimulated important growth pathways including angiogenesis. Preconditioning the decellularized tissue with MMPs before recellularization increased cell density by 200–300% compared with standard culturing techniques and improved the cell distribution, independent of the decellularization method used for scaffold generation. Hence, our findings suggest that decellularization protocol-dependent deficiencies may be compensated for by enzymatic preconditioning. In addition, the recellularization platform and culturing technique play important roles in order to obtain cell-rich bioengineered constructs suitable for in vivo applications.

## Methods

### Uterus isolation and decellularization

Eighteen sheep uteri of about 35 g each were isolated from 8- to 12-month-old Swedish Finull sheep at a local abattoir. Since the animals were processed for food production, no prior animal welfare evaluation was required for the organ procurement. Each uterus was cannulated, flushed, and decellularized by perfusion through the organ vasculature according to three previously characterized and published protocols^30^. In brief, P1 was based on the decellularization detergent SDS (0.5%), P2 on SDC (2%), and P3 on a combination of SDC (2%) and Triton X-100 (1%). Each detergent in P1 and P2 was perfused for 8 h, respectively, which was then followed up by the perfusion of deionized water (DW; 26 h), phosphate buffer saline (PBS; 12 h), DNase I (1 h; 37 °C; 8000IU per organ) and then another hour with DW. This cycle was then repeated a second time. For P3, the initial detergent perfusion with SDC lasted only 4 h, which was then followed up by the perfusion of DW (6 h), Triton X-100 (12 h), and then DW (36 h). This cycle was then repeated a second time before the organs in this group were exposed to a 1 h DNase I treatment at 37 °C (8000IU per organ). All organs from P1 to P3 were then washed with DW for an additional 48 h, then sterilized by the perfusion of peracetic acid (1 h, 0.1% in normal saline). The organs were then washed for 48 h in sterile PBS and frozen at −20 °C until used for the experiments mentioned below. The physical attributes of these uterus scaffolds were extensively documented in our earlier publication and were therefore not further assessed herein. To standardize each experiment below, a biopsy punch was used to create multiple full-thickness dUTDs (5 mm in diameter) that were obtained from the uterine body, a few cm proximal to the cervix. Thus, each dUTD contained all uterine layers. All dUTDs were then kept in PBS until used for the experiments described below.

### Scaffold MMPs preconditioning, DNA, protein, and hydroxyproline quantification

About half of all the dUTDs from each scaffold type were enzymatically treated with MMP2 (2.5 μg/L, Peprotech, Stockholm, Sweden) and MMP9 (2.5 μg/L, Sigma-Aldrich, Gothenburg, Sweden) that had been dissolved and activated according to Sigma-Aldrich instructions using aminophenylmercuric acetate buffer. Each scaffold type was incubated at 37 °C for 24 h in the MMP solution. Each disc was then washed with 20 mM EDTA solution twice, and then with PBS.

Six dUTDs from each scaffold type, including MMPs treated, were analyzed for donor DNA content using the DNeasy Blood and Tissue Kit (#69504; Qiagen, Sollentuna, Sweden) and a Nanodrop 2000 (Thermo Fisher Scientific, Gothenburg, Sweden). The total protein scaffold content (*n* = 6 per group) was established using the Coomassie Bradford protein assay kit as per standard protocols (#23200; Thermo Fisher Scientific).

Hydroxyproline, the main constituent in collagen, was quantified from 100 mg of homogenized tissue (*n* = 6 per group). Homogenates were first mixed with NaOH (10 N) and incubated for 1 h at 120 °C. The hydroxyproline content was then measured using a hydroxyproline assay kit (ab222941, Abcam, Cambridge, MA) following the manufactures instructions.

### Histology and immunohistochemistry assessment on scaffolds

Five µm thick tissue sections from dehydrated and paraffin-embedded MMPs treated and untreated dUTDs were mounted on slides and processed for Russel-Movat pentachrome staining using a standard staining kit (KSC-MPS-2, Nordic BioSite, Täby, Sweden). Sections from each scaffold type were also processed for immunohistochemistry. An antigen retrieval step was first conducted using citric acid (pH = 6.0) in a pressure cooker. The sensitive Mach 3 and vulcan fast red kits (Biocare Medical, Pacheco, CA, USA) were then used to visualize collagen I (#ab292), collagen IV (#ab6586), laminin (#ab11575), elastin (#ab23748), and fibronectin (#ab6328) using antibodies from Abcam (Cambridge, UK) at a concentration of 1:100.

### Vascular conduit patency test after MMPs treatment

The Batson’s #17 plastic replica corrosion kit (Polysciences, Eppelheim, Germany) was used to investigate if the MMPs treatment may have negatively affected the patency of the vascular conduits in the scaffolds. Large scaffolds that represented half a sheep uterus with remaining vasculature were cannulated in the uterine artery and perfused with 5 ml of the same MMP solution as used for the dUTDs. The perfused decellularized half sheep uterus was then submerged in the same solution in a 50 ml Falcon tube and incubated at 37 °C for 24 h. Each scaffold was then perfused and washed with 20 mM EDTA solution twice and then with PBS. Polymer resin from the Batson’s kit was mixed according to the instructions and was then immediately injected into the uterine artery. The procedure was filmed and the files attached as supplementary information (Supplementary videos 1–3).

### Functional bioactivity assessment of the different uterus scaffold types

Two bioactivity tests were conducted on each scaffold type: the DRG assay which is responsive to growth factors that stimulate axonal regeneration^58^, and the chicken CAM assay which is responsive to angiogenic growth factors^59,60^.

#### The DRG assay

Bilateral DRGs were isolated from embryonic day 14 fetuses of a pregnant Wistar rat (Janvier Labs, Le Genest-Saint-Isle, France) following approved ethical guidelines (114–2014, University of Gothenburg, Sweden). Isolated DRGs were immediately placed separately on top of the endometrial side of dUTDs (*n* = 8/protocol). These co-cultures were incubated for 2 days in standard cell culturing medium (DMEM GlutaMAX, 1% Anti-Anti, 10% fetal bovine serum, FBS; Thermo Fisher Scientific). DRGs cultured in collagen-coated wells with media supplemented with nerve growth factor (NGF, 10 ng/mL; PeproTech, Stockholm, Sweden) were used as positive controls. Neuronal outgrowth was assessed by fluorescent immunocytochemistry on paraformaldehyde fixed co-cultures using an anti-pan neurofilament primary antibody (1:400, AB837904, Nordic Biosite, Täby, Sweden) and an Alexa Fluor 488 conjugated secondary antibody (1:300; Thermo Fisher Scientific). DNA was counterstained with 4′,6′-diamidino-2-phenylindole (DAPI, Thermo Fisher Scientific).

#### The chicken CAM assay

Fertilized chicken eggs (Swedfarm Väst AB, Herrljunga, Sweden) were placed in an egg incubator for 3 days at 37.5 °C and then 3 mL of albumin was removed using a sterile 25 G needle. This detached the CAM from the shell, and then a 1 cm^2^ window was cut in the shell. The opening was covered with Tegaderm film (3 M Health Care, Minneapolis, Minnesota, USA) before each egg was returned to the incubator. On embryo development day (EDD) 9, the window was re-opened and a dUTD was inserted onto the CAM (P1, *n* = 5; P1_MMPs_, *n* = 6; P2, *n* = 6; P2_MMPs_, *n* = 4; P3, *n* = 6; P3_MMPs_, *n* = 5). A control group was included to evaluate normal blood vessel growth by inoculating a biologically inert reference site on the CAM using a hydrogel drop of 2.2% alginate/PBS (*n* = 7; 20 μL). The window was again covered with Tegaderm and the egg placed back in the incubator. On EDD 14, the shell was opened further to enable photographing the implant and the embryonic vessels on the CAM under a stereomicroscope. From the photograph of each implant, two rings (0.5–1.5 mm from the center) were digitally inserted in the picture at the biopsy center using ImageJ. The number of vessels was then manually counted in the area between these rings (branching vessels were counted as one) by two individuals, one of whom was blinded to the study. The inter-observer variation was insignificant, and thus, counts were averaged and used for the statistical analyses.

### Sheep fetal stem cell isolation and characterization

Heterogeneous SF-SCs were isolated from the femurs of a 6–8-week-old sheep fetus (obtained at the local abattoir) by flushing with L-15 medium supplemented with Anti-Anti (Thermo Fisher Scientific). Cells were then expanded in vitro and verified for multipotency according to differentiation methods for chondrogenesis^61^, osteogenesis^62^, and myogenesis^63^. Differentiated and undifferentiated SF-SCs were fixed in 4% buffered formaldehyde and stained with a wide range of primary antibodies (1 h at room temperature) from Abcam (Amsterdam, Netherlands), including α-SMA (ab32575; 1:500), vimentin (ab8798; 1:100), CD166 (ab235957; 1:200), Ki67 (ab15580; 1:300), estrogen receptor-α (ER-α; ab66102; 1:100), ER-β (ab187291; 1:100), progesterone receptor (PR; ab2765; 1:100), cytokeratin (ab9377; 1:1000), MyoD1 (ab16148, 1:100), RANK (ab13918, 1:100), and DMP1 (ab103203, 1:100). Each primary antibody was conjugated with either CY3 or Alexa Fluor 488 secondary antibodies (Thermo Fisher; 1:300) and DNA was labeled with DAPI. Chondrogenesis was evaluated after alcian blue staining using standard methods.

### Recellularization using standard cell culturing conditions

The dUTDs were recellularized with SF-SCs by injecting each disc (*n* = 20 per scaffold type) with a total of 10^6^ cells in 10 repeated injections using a 30 G needle. The recellularized scaffolds were cultured with the endometrial side positioned upwards using standard cell culturing conditions with medium changed every second day. This group was named RC_SC_.

### Recellularization using transwell inserts

Additional dUTDs (*n* = 20 per scaffold type) were placed individually on transwell inserts (0.4 μm, Sarstedt, Nümbrecht, Germany) in a 24-well plate and were then completely covered with cell culture medium. The transwell inserts improved cell culture medium access to the bottom (myometrial side) of the recellularized scaffold during culture and thereby also the nutritional and metabolite exchange in the deeper compartment of the scaffolds. The cell injections were conducted the same way as mentioned above. This group was named RC_TW_.

### Recellularization of enzymatically preconditioned scaffolds in transwell inserts

Twenty dUTDs per scaffold type were placed individually in transwell inserts and recellularized and cultured as earlier mentioned. This group was named RC_MMP+TW_.

### Histology, immunohistochemistry, and cell counting on recellularized scaffolds

The recellularization efficiency of each construct was evaluated on day 3 and day 14 post recellularization day by histology using standard hematoxylin & eosin (H&E) and DAPI staining protocols (*n* = 9 per scaffold type, per recellularization method, and per time point). In addition, some sections were stained with the same antibodies that were used to characterize the cells in vitro, using the same methods but with an additional antigen retrieval step using citrate buffer. The recellularization efficiency (cells/mm^2^) was based on the total number of DAPI-positive cells found in six randomly photographed fields from each recellularized dUTD (×200 magnification; photo area = 0.776 mm^2^). The total cell number was quantified using ImageJ, then the value transformed to number of cells/mm^2^, and was then averaged for each group, recellularization strategy, scaffold type, and time point after recellularization.

### Scanning electron microscopy

Samples assessed by scanning electron microscopy included discs of decellularized-, MMPs treated-, and recellularized tissue. Each specimen was prepared using the osmium tetroxide and thiocarbohydrazide method^64^, and was imaged with Zeiss Gemini-2 SEM 500 (Carl Zeiss AG, Oberkochen, Germany). The quantification of collagen fiber thickness was performed on images taken at ×50,000 magnification. Each image was opened in ImageJ, the scale was set and the measuring tool in the software was used to measure the width of 25 individual collagen fiber strands in each image.

### Statistics

Statistics was performed using Graphpad Prism (Graphpad, CA, USA). All data sets were tested for normality using the Shapiro–Wilk test. Parametric data were assessed with one-way ANOVA and Tukey’s HSD post hoc test and presented with bar graphs (mean ± standard error of mean, SEM). Non-parametric data were presented in box plots with median, interquartile range, and range. Kruskal–Wallis test was conducted to evaluate differences (corrected by Dunn’s post hoc test for multiple group comparisons). Mann–Whitney U-test was used to compare differences between the recellularized constructs after 3 and 14 days in vitro for the same scaffold type and culturing condition.

### Reporting summary

Further information on research design is available in the Nature Research Reporting Summary linked to this article.

## Supplementary information

Supplementary video legends

Supplementary video 1

Supplementary video 2

Supplementary video 3

Reporting Summary

## Data Availability

Original data generated for this article are available from the corresponding author on reasonable request.
