# Supplementary material for: Towards a bioengineered uterus: bioactive sheep uterus scaffolds are effectively recellularized by enzymatic preconditioning
Source: NPJ Regen Med. 2021 May 21;6:26. doi: 10.1038/s41536-021-00136-0 (PMC8140118; doi:10.1038/s41536-021-00136-0)
Supplement: Supplementary file 1 — Supplementary video legends [file 41536_2021_136_MOESM1_ESM.docx]

**Supplementary video legends**

**Supplementary video 1.** A half sheep uterus scaffold produced by protocol 1 was perfused through the uterine artery with Batson’s #17 after it was preconditioned by matrix metalloproteinase 2 and 9 (MMPs) that confirmed its vascular conduit patency.

**Supplementary video 2.** A half sheep uterus scaffold produced by protocol 2 was perfused through the uterine artery with Batson’s #17 after it was preconditioned by matrix metalloproteinase 2 and 9 (MMPs) that confirmed its vascular conduit patency.

**Supplementary video 3.** A half sheep uterus scaffold produced by protocol 3 was perfused through the uterine artery with Batson’s #17 after it was preconditioned by matrix metalloproteinase 2 and 9 (MMPs) that confirmed its vascular conduit patency.
