# Supplementary material for: Towards a bioengineered uterus: bioactive sheep uterus scaffolds are effectively recellularized by enzymatic preconditioning
Source: NPJ Regen Med. 2021 May 21;6:26. doi: 10.1038/s41536-021-00136-0 (PMC8140118; doi:10.1038/s41536-021-00136-0)
Supplement: Supplementary file 5 — Reporting Summary [file 41536_2021_136_MOESM5_ESM.pdf]

nature research

Corresponding author(s): Mats Hellström

Last updated by author(s): 9 April, 2021

Reporting Summary

Nature Research wishes to improve the reproducibility of the work that we publish. This form provides structure for consistency and transparency in reporting. For further information on Nature Research policies, see our Editorial Policies and the Editorial Policy Checklist.

Statistics

For all statistical analyses, confirm that the following items are present in the figure legend, table legend, main text, or Methods section.

n/a	Confirmed	
☐	☒	The exact sample size (n) for each experimental group/condition, given as a discrete number and unit of measurement
☐	☒	A statement on whether measurements were taken from distinct samples or whether the same sample was measured repeatedly
☐	☒	The statistical test(s) used AND whether they are one- or two-sided<br>*Only common tests should be described solely by name; describe more complex techniques in the Methods section.*
☐	☒	A description of all covariates tested
☐	☒	A description of any assumptions or corrections, such as tests of normality and adjustment for multiple comparisons
☐	☒	A full description of the statistical parameters including central tendency (e.g. means) or other basic estimates (e.g. regression coefficient) AND variation (e.g. standard deviation) or associated estimates of uncertainty (e.g. confidence intervals)
☒	☐	For null hypothesis testing, the test statistic (e.g. F, t, r) with confidence intervals, effect sizes, degrees of freedom and P value noted<br>*Give P values as exact values whenever suitable.*
☒	☐	For Bayesian analysis, information on the choice of priors and Markov chain Monte Carlo settings
☒	☐	For hierarchical and complex designs, identification of the appropriate level for tests and full reporting of outcomes
☒	☐	Estimates of effect sizes (e.g. Cohen's d, Pearson's r), indicating how they were calculated

Our web collection on statistics for biologists contains articles on many of the points above.

Software and code

Policy information about availability of computer code

Data collection	Cell counts were automated using the imageJ software.
Data analysis	Graphpad Prism 9.1

For manuscripts utilizing custom algorithms or software that are central to the research but not yet described in published literature, software must be made available to editors and reviewers. We strongly encourage code deposition in a community repository (e.g. GitHub). See the Nature Research guidelines for submitting code & software for further information.

Data

Policy information about availability of data

All manuscripts must include a data availability statement. This statement should provide the following information, where applicable:

- Accession codes, unique identifiers, or web links for publicly available datasets
- A list of figures that have associated raw data
- A description of any restrictions on data availability

Original data generated for this article are available from the corresponding author on reasonable request.

Field-specific reporting

Please select the one below that is the best fit for your research. If you are not sure, read the appropriate sections before making your selection.

☒ Life sciences ☐ Behavioural & social sciences ☐ Ecological, evolutionary & environmental sciences

For a reference copy of the document with all sections, see nature.com/documents/nr-reporting-summary-flat.pdf

Life sciences study design

All studies must disclose on these points even when the disclosure is negative.

Sample size	No sample-size calcuation was performed.
Data exclusions	No data exclusions were done
Replication	The recellularization experiment was repeated three sperate times with an n=3 for each time point. The n-tot for these experiments are presented in the graphs in figure six as n=9.
Randomization	N/A
Blinding	Two persons were blinded to the groups for the quantification of the CAM assay that is presented in figure four. The person who quantified the cells after recellularization was not blinded to the study groups, but the cell quantifications were conducted automatically using imageJ software. The remaning quantifications were kit-based and the labwork was not conducted blinded for these quantifications since the data was collected from messurments done by a machine.

Reporting for specific materials, systems and methods

We require information from authors about some types of materials, experimental systems and methods used in many studies. Here, indicate whether each material, system or method listed is relevant to your study. If you are not sure if a list item applies to your research, read the appropriate section before selecting a response.

Materials & experimental systems

n/a	Involved in the study	
☐	☒	Antibodies
☐	☒	Eukaryotic cell lines
☒	☐	Palaeontology and archaeology
☐	☒	Animals and other organisms
☒	☐	Human research participants
☒	☐	Clinical data
☒	☐	Dual use research of concern

Methods

n/a	Involved in the study	
☒	☐	ChIP-seq
☒	☐	Flow cytometry
☒	☐	MRI-based neuroimaging

Antibodies

Antibodies used	Collagen I (#ab292), collagen IV (#ab6586), laminin (#ab11575), elastin (#ab23748) and fibronectin (#ab6328) using antibodies from Abcam (Cambrige, UK) at a concentration of 1:100.<br>Anti-pan neurofilament primary antibody (1:400, AB837904, Nordic Biosite, Täby, Sweden) and an Alexa Fluor 488 conjugated secondary antibody (1:300; Thermo Fisher Scientific).<br>Abcam (Cambrige, UK) antibodies for α-SMA (ab32575; 1:500), vimentin (ab8798; 1:100), CD166 (ab235957; 1:200), Ki67 (ab15580; 1:300), estrogen receptor-α (ER-α; ab66102; 1:100), ER-β (ab187291; 1:100), progesterone receptor (PR; ab2765; 1:100), cytokeratin (ab9377; 1:1000), MyoD1 (ab16148, 1:100), RANK (ab13918, 1:100) and DMP1 (ab103203, 1:100) were also used. For immunofluorescence, each primary antibody was conjugated with either CY3 or Alexa Fluor 488 secondary antibodies (Thermo Fisher; 1:300).
Validation	All antibody stainings were validated by a negative control where the primary antibody had been replaced by diltuion solution.

Eukaryotic cell lines

Policy information about cell lines

Cell line source(s)	Primary fetal sheep stem cells isolated in-house.
Authentication	Cells were cherecterized in-house and the data is presented in the manuscript as figure five.

Mycoplasma contamination	Was not tested for mycoplasma
Commonly misidentified lines (See ICLAC register)	N/A

Animals and other organisms

Policy information about studies involving animals; ARRIVE guidelines recommended for reporting animal research

Laboratory animals	Pregnant Wistar rat (Janvier labs, Le Genest-Saint-Isle, France) following approved ethical guidelines (114-2014, University of Gothenburg, Sweden).
Wild animals	N/A
Field-collected samples	N/A
Ethics oversight	All experiments followed the approved guidlines stated in the ethics document 114-2014. Approval was given by the local wellfare committee at University of Gothenburg, Sweden.

Note that full information on the approval of the study protocol must also be provided in the manuscript.
